# Angioedème médicamenteux: un effet secondaire inhabituel de la simvastatine

**DOI:** 10.11604/pamj.2017.26.213.5876

**Published:** 2017-04-19

**Authors:** Abdelilah Ben El Mekki, Ali Chaib

**Affiliations:** 1 Cardiology Department, Military Training Hospital Mohammed V, Rabat, Maroc

**Keywords:** Angiœdème, simvastatine, effet secondaire, Drug induced angioedema, simvastatin, side effects

## Image en médecine

L'angioedème histaminique médicamenteux, est une urgence diagnostique et thérapeutique car le pronostic vital peut être mis en jeu à court terme par le risque d’évolution vers un œdème de Quincke. Il se définit par un gonflement soudain localisé non inflammatoire des tissus sous cutanés et/ou sous-muqueux, il se voit surtout avec les pénicillines, les produits de contraste iodés, les inhibiteurs de l'enzyme de conversion. Il est rarement observé avec la simvastatine. Nous rapportons le cas d’une patiente âgée de 43 ans, suivie pour hypercholestérolémie sous régime depuis deux ans mal équilibrée, a présenté 24h après la prise de la simvastatine un œdème non inflammatoire au niveau des paupières et des joues associé à une macrochéilite avec une sensation de tension douloureuse, sans lésions urticariennes ni dysphonie ni dyspnée, le tout évoluant dans un contexte de conservation de l’état général. La patiente a été hospitalisée avec arrêt de toute médication, elle a reçu une injection d’hémissuccinate d’hydrocortisone et d’antihistaminique. L’évolution était favorable, une disparition de l’angioedème était observée 48h après le traitement. Une déclaration au centre de pharmacovigilance et une interdiction de la réintroduction des statines était réalisée.

**Figure 1:**
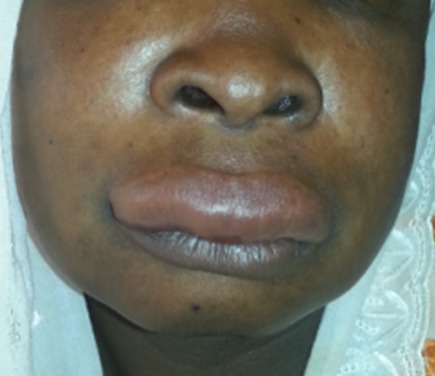
oedème non inflammatoire au niveau des paupières associée à une macrochéilite

